# Disease named entity recognition from biomedical literature using a novel convolutional neural network

**DOI:** 10.1186/s12920-017-0316-8

**Published:** 2017-12-28

**Authors:** Zhehuan Zhao, Zhihao Yang, Ling Luo, Lei Wang, Yin Zhang, Hongfei Lin, Jian Wang

**Affiliations:** 1 College of Computer Science and Technology, Dalian University of Technology, Dalian, 116023 China; 2 Beijing Institute of Health Administration and Medical Information, Beijing, 100850 China

**Keywords:** Disease, Named entity recognition, Convolutional neural network, Deep learning multiple label strategy

## Abstract

**Background:**

Automatic disease named entity recognition (DNER) is of utmost importance for development of more sophisticated BioNLP tools. However, most conventional CRF based DNER systems rely on well-designed features whose selection is labor intensive and time-consuming. Though most deep learning methods can solve NER problems with little feature engineering, they employ additional CRF layer to capture the correlation information between labels in neighborhoods which makes them much complicated.

**Methods:**

In this paper, we propose a novel multiple label convolutional neural network (MCNN) based disease NER approach. In this approach, instead of the CRF layer, a multiple label strategy (MLS) first introduced by us, is employed. First, the character-level embedding, word-level embedding and lexicon feature embedding are concatenated. Then several convolutional layers are stacked over the concatenated embedding. Finally, MLS strategy is applied to the output layer to capture the correlation information between neighboring labels.

**Results:**

As shown by the experimental results, MCNN can achieve the state-of-the-art performance on both NCBI and CDR corpora.

**Conclusions:**

The proposed MCNN based disease NER method achieves the state-of-the-art performance with little feature engineering. And the experimental results show the MLS strategy’s effectiveness of capturing the correlation information between labels in the neighborhood.

## Background

The recognition of disease named entities automatically from biomedical literature is of utmost importance as it is the foundation of other more sophisticated NLP tools such as information extraction, question answering, text summarization etc. [1]. As reported in [2], complicate and inconsistent terminologies, new disease names, multiple names for the same disease, complex syntactic structure referring to multiple related names or mentions are some of the major reasons for making automatic disease named entity recognition (DNER) task challenging. Therefore, most state-of-the-art conventional CRF based DNER systems [3–6] have to design much complicate features (lexical features, syntactic features, semantic features, morphological features, dictionary features, embedding features, terminology features, vowel features, etc.) manually which not only requires linguistic and domain insight but also is time consuming.

Recently, many deep learning based methods were proposed to solve the NER problems of general field and they achieved the state-of-the-art performance with little feature engineering. Collobert et al. [7] proposed a simple but effective feed-forward neutral network method to solve the sequence tagging problem. They introduced a sentence level log-likelihood to consider the correlation information between labels in neighborhoods. Later, Santos and Guimaraes [8] extended Collobert et al.’s method with character-level representation to extract the morphological information (like the prefix or suffix of a word) from characters of words. The character-level representation was obtained using convolutional neural network (CNN) [9]. Chiu and Nichols [10] proposed a hybrid of bidirectional LSTM (BLSTM) [11] and CNN to model both word-level and character-level representations. Similar to [8], CNN was employed to encode character-level information of a word into its character-level representation. Then the word-level and character-level representations were combined and fed into a BLSTM. They also utilized the sentence level log-likelihood, reported in [7], to decode the labels of a sentence jointly. Ma and Hovy proposed LSTM-CNN-CRF approach to solve sequence labeling problems end-to-end [12]. This approach is almost the same as the approach represented in [10] except the labels’ decoding process in which a sequential CRF is utilized to jointly decode labels for the whole sentence. Different with the deep learning methods above, Lample et al. [13] proposed a BLSTM-CRF model in which the character-level representation was extracted using BLSTM instead of CNN.

Compared with the deep learning based methods in the general field, few deep learning methods were applied to the disease NER problems. Sahu and Anand [14] proposed the various recurrent neural networks (RNNs) [15] based disease name recognition model which achieved the state-of-the-art performance on NCBI disease corpus [4]. Their approach is similar to that of [11] and the main difference between them is that additional features (i.e., character-type, capitalization and lexicon features) are used in latter but not in the former.

Currently, the following two problems exist in the disease NER research. First, most of the state-of-the-art conventional CRF based methods rely heavily on task-specific feature engineering that limits their generalization ability. Second, most deep learning methods treat NER as a sentence level sequence tagging problem. Thus, frequently, a decoding layer (like CRF) is adopted to decode the labels of a sentence jointly which makes it more complicate than it should be. Since, the transition probability matrix (parameters of the decoding layer) should be learned additionally and another decoding process (searching for the optimal label sequence using Viterbi algorithm [16]) should be conducted.

To solve the above problems, a novel deep learning based disease NER architecture, i.e., multiple label convolutional neural network (MCNN), is introduced in our method. We assume that the context information of the target word is enough for predicting the target word’s label correctly. Therefore, MCNN treats NER as a word level classification problem in which only the information of words to a fixed-size window around the target word is fed into MCNN. Then, the target word is classified into one of the three labels including “B”, “I” and “O”, as the BIO tagging scheme is employed in our experiments. Similar to the other state-of-the-art deep learning methods, MCNN needs little feature engineering. What’s more, compared with other deep learning methods, it is easier to implement.

First, MCNN needs little feature engineering. Besides the word-level and character-level embeddings, only the lexicon feature embedding is employed as input. Among them, the character-level embedding and the lexicon feature embedding are initialized randomly and the word-level embedding is initialized with the pre-trained embedding using Word2vec [17]. Then these embeddings will be tuned automatically through the training process. Therefore, MCNN needs no hand-crafted features except the lexicon feature. Second, instead of the CRF layer, multiple label strategy (MLS) is first introduced to capture the correlation information between labels in neighborhoods by predicting the previous and the next words’ labels in auxiliary. MLS is implemented by enlarging the output layer’s size which is much easier than the implementation of a CRF layer. Finally, with little feature engineering and simple implementation, MCNN achieves the state-of-the-art performance on both NCBI corpus [4] and CDR corpus [18].

## Methods

**Fig. 1:**
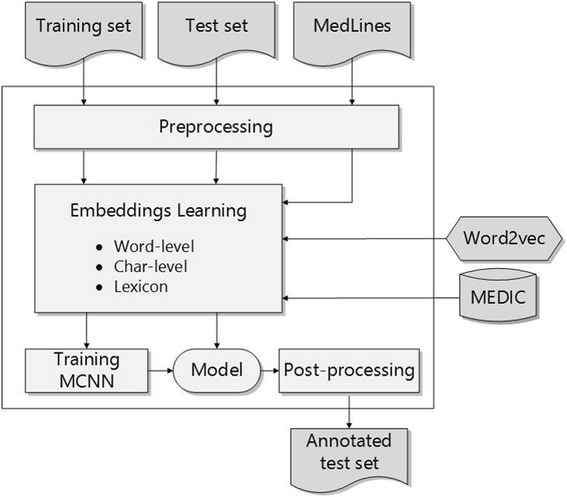
The processing flow of our method

MCNN contains four processing steps as shown in Fig. 1.

1. Preprocessing step which constructs an easily understood corpus for MCNN.
2. Embeddings learning step that initializes various embeddings using Word2vec and MEDIC [19].
3. Training MCNN model step that learns a classifier based on the above initialized embeddings.
4. Post-processing step that regulates the predicted results to improve the final performance.

The details are described in the following sections.

### Preprocessing

Appropriate preprocessing can boost the final performance significantly. Therefore, in our method, two preprocessing operations (i.e. tokenization and transforming the numbers to uniform form) are conducted.

#### Tokenization

Since tokenization process is one of the standard preprocessing steps, it is performed in our method as well. The aim of tokenization is to split the sentence into atomic units and we simply tokenize each sentence using space and characters in “/−− > <;:?[]{}()!@#$%^&* − +”.

#### Transforming the numbers to uniform form

Numbers (integers and decimals) occur frequently in the biomedical literature. For example, in the sentence “164 patients (mean age +/- standard deviation [SD] 81.6 +/- 6.8 years) were admitted”, there are one integer (164) and two decimals (“16.6” and “49.1”). Transforming them to a uniform form (“num”) won’t change the sentence’s semantic expression. Therefore, the sentence becomes “num patients (mean age +/- standard deviation [SD] num +/- num years) were admitted”. Then we train a word embedding on the processed sentence with Word2vec and it will generate an embedding for “num” instead of for “164”, “81.6” and “6.8”. Since Word2vec trains a sentence based on sliding window mechanism, the “num” will be trained three times while “164”, “81.6” and “6.8” are trained once only. As more training times will generate more accurate embedding, replacing all the integers and decimals with “num” will provide more powerful embedding for “num”. In addition, it will significantly reduce the size of the vocabulary and make the embedding more compact.

### Embeddings learning

In our method, each word is represented as a real vector which is generated by concatenating corresponding word-level embedding, character-level representation and lexicon feature embedding. Later, these three embeddings will be described in details in the following sections. In addition, a dropout layer [20] is adopted after the concatenation process.

#### Word-level embedding

A word embedding is a parameterized function that maps words to high-dimensional vectors. Word embedding was firstly introduced in [21] to fight the curse of dimensionality in the process of learning language model using neural network. Most deep learning based NER methods take the word embeddings as the fundamental input [7, 8, 10, 12–14]. Since the larger corpus will generate the better embedding [22], besides the original CDR and NCBI corpora, a total of 2,008,726 Medline abstracts were downloaded from PubMed website (http://www.ncbi.nlm.nih.gov/pubmed/) to learn the word embedding with a query string “disease”. After the preprocessing step, these processed Medline abstracts and the disease corpora are fed into Word2vec to learn the initial values of the word-level embedding.

#### Charactor-level representation

**Fig. 2:**
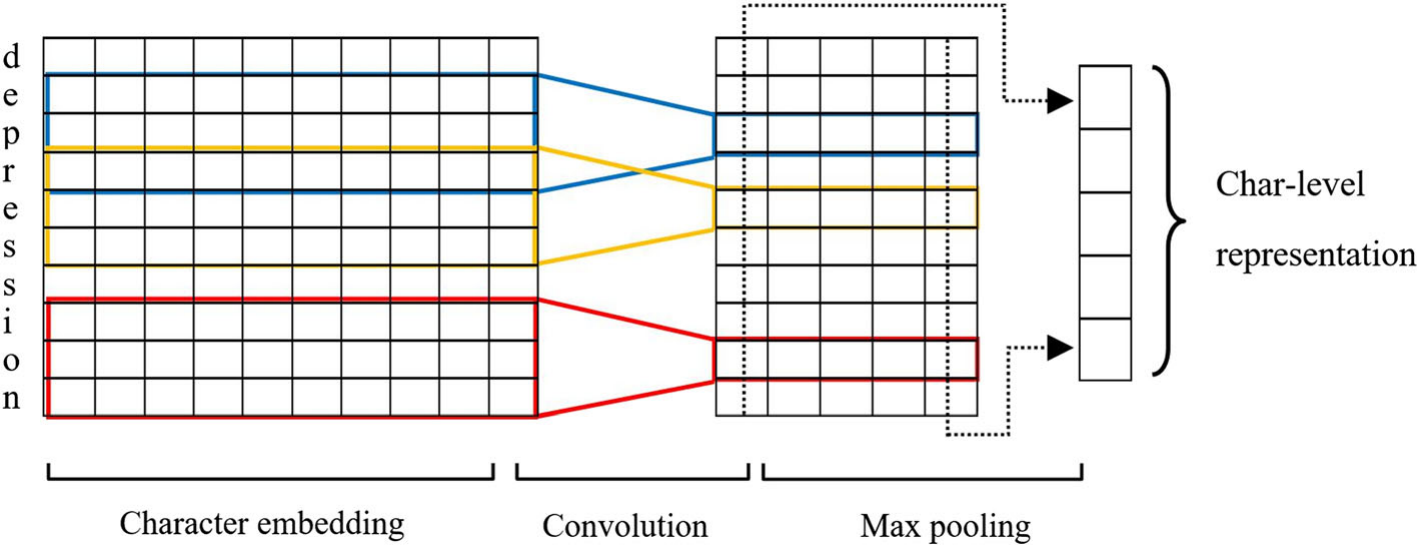
Generation of the character-level representation using convolutional neural network

It has been proved [8, 10, 12, 14] that CNN is an effective method to extract morphological information (like the prefix or suffix of a word) from characters of a word. It also could be useful with rare words whose embedding values are poorly trained. Therefore, we also employ a CNN to extract the character-level representation of a given word, which is shown in Fig. 2. First, each character of a word is projected to a real vector using the character lookup table. Then, a convolutional layer and a max-pooling layer are adopted orderly. In addition, a dropout layer [20] is applied after the projecting process. The character lookup table is initialized randomly to output a vector of 20 dimensions and the character set includes all unique characters in the CDR and NCBI corpora.

#### Lexicon feature embedding

Most state-of-the-art disease NER systems [5, 6, 14] benefit from integrating domain resources as a form of external knowledge. In our method, MEDIC [19] is employed to extract the lexicon feature embedding. MEDIC [19] is both a deep and broad vocabulary, composed of 9700 unique diseases described by more than 67,000 terms (including synonyms), which is created by merging and combining the best two disease sources (OMIM [23] and MeSH’s “Disease” branch [24]).

In MCNN, the lexicon feature embedding is learned by following two steps: First, dictionary matching process is conducted using MEDIC on both training and test sets. Then, each word will be tagged as one of the labels (“B”, “I”, and “O”). Second, the tagged labels are projected to the corresponding real vectors using the lexicon lookup table that is initialized randomly.

### Training MCNN

Different from other state-of-the-art deep learning methods which regard NER as a sentence level sequence tagging problem, MCNN treats NER as a simple word-level classification problem. We assume that the label of a word depends mainly on the neighboring words instead of the whole sentence. Therefore, multiple convolutional layers, instead of the RNNs (e.g. LSTM) that are more suitable for sequence labeling problems, are employed to capture the context information. However, predicting each word’s label independently will miss the dependency information between labels (e.g. the label “O” should never be followed by a label “I”). This problem used to be solved by adding additional decoding layer (e.g. CRF), which makes it more complex and inefficient. Since the transition probability matrix (parameters of the decoding layer) should be learned additionally and another decoding process using Viterbi algorithm is needed. Therefore, instead of the CRF layer, MLS, first introduced in our method, is adopted in MCNN. MLS captures the correlations between labels in neighborhoods easily by predicting the neighboring words’ labels in auxiliary (i.e. predicting the current, the previous and the next words’ labels simultaneously). MLS is implemented by enlarging the output layer’s size which is much easier than the implementation of the CRF that needs learning additional parameters and decoding the whole sentence labels jointly with Viterbi algorithm.

**Fig. 3:**
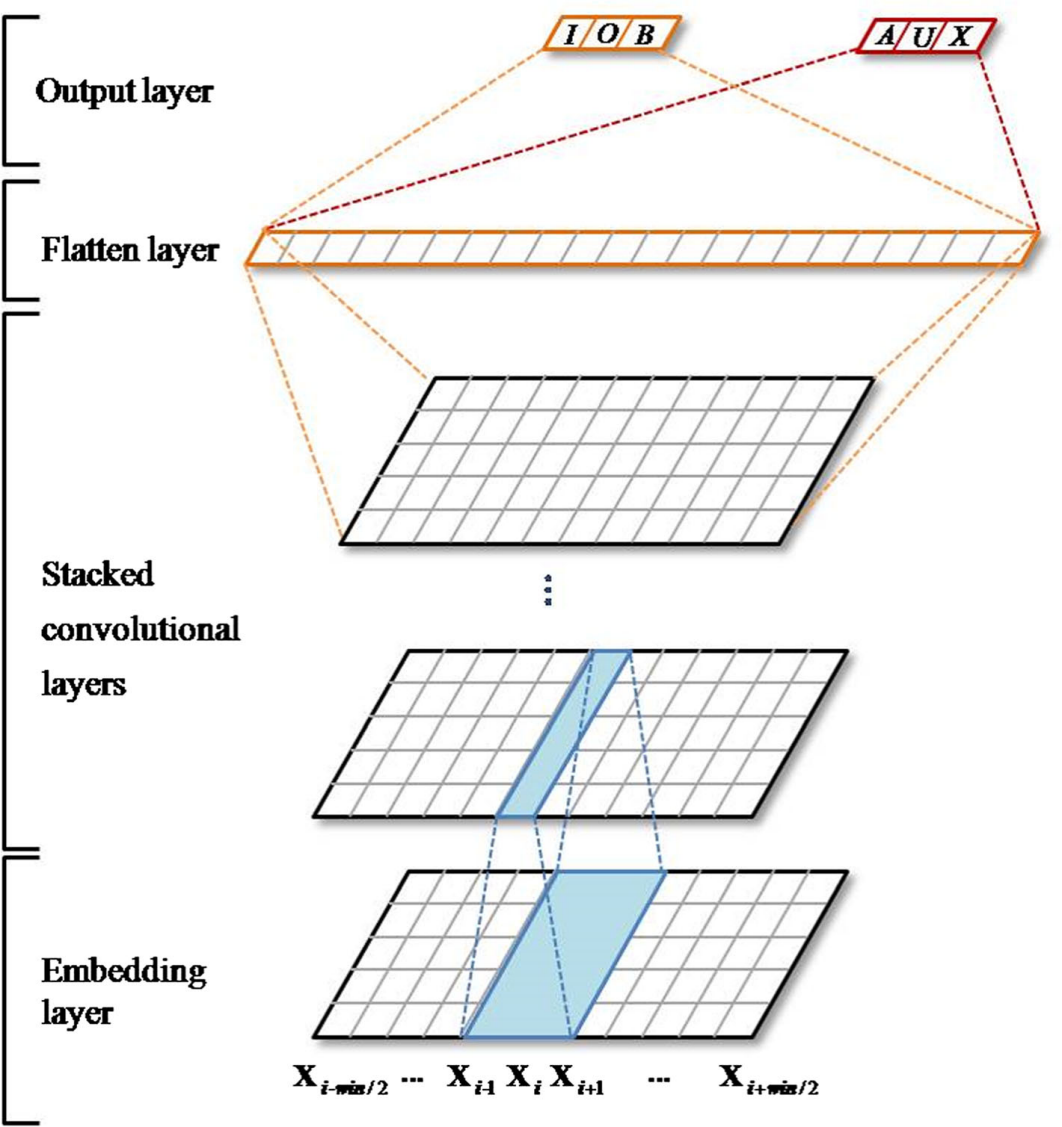
The architecture of MCNN

The details of the MCNN architecture are shown in Fig. 3. Given a word (Xi), the fixed-size window of words around it are input into the MCNN model. First, each word is represented as a real vector by concatenating the corresponding word-level embedding, character-level representation and lexicon feature embedding. Then, several convolutional layers are stacked over the real vector to extract the higher level features. After the last convolutional layer, a flatten layer is followed with which all the vectors (outputs of the last convolutional layer) are concatenated to generate a larger one. Finally, the full-connected output layer is stacked over the flatten layer and obtains the output vectors: ${\mathbf{out}}^{\mathbf{m}\mathbf{ain}}=\left[{\mathbf{out}}_{\mathbf{1}}^{\mathbf{m}},{\mathbf{out}}_{\mathbf{2}}^{\mathbf{m}},\dots, {\mathbf{out}}_{\mathbf{z}}^{\mathbf{m}}\right]$ and ${\mathbf{out}}^{\mathbf{a}\mathbf{ux}}=\left[{\mathbf{out}}_{\mathbf{1}}^{\mathbf{a}},{\mathbf{out}}_{\mathbf{2}}^{\mathbf{a}},\dots, {\mathbf{out}}_{\mathbf{k}}^{\mathbf{a}}\right]$ where ${\mathbf{out}}_{\mathbf{i}}^{\mathbf{m}}$ and ${\mathbf{out}}_{\mathbf{i}}^{\mathbf{a}}$ stand for the confidence scores of the corresponding labels for the main and auxiliary outputs, respectively.

As can be seen from Fig. 3, there is no pooling layer (e.g., max-pooling, average-pooling) in the MCNN architecture, while the convolutional layer is usually employed together with a pooling layer [9, 25–28]. The pooling layer mainly aims to achieve shift-invariance by reducing the resolution of the feature maps and also can lower the computational burden by reducing the number of connections between convolutional layers [29]. However, the shift-invariance property is vital for the image-relevant tasks but not for the text-relevant problems. And our method’s computation complexity is much lower than that of other deep learning methods, as only a limited context is considered as input instead of the whole sentence. What’s more, pooling layers likely lead to the loss of information [30]. Therefore, no pooling layer is adopted in MCNN.

We define *θ*as all the parameters of our model. Then the probability value of each label is obtained through the following softmax operation over all possible labels.

1 $$ {p}_{main}\left(i|x,\theta \right)=\frac{e^{out_i^m}}{\sum_{j=1}^z{e}^{out_j^m}} $$

2 $$ {p}_{aux}\left(i|x,\theta \right)=\frac{e^{out_i^a}}{\sum_{j=1}^k{e}^{out_j^a}} $$

Then the log likelihood of the parameters is calculated as follows when all training instances ($T=\left\{{x}^{(i)},{y}^{(i)},{y}_{aux}^{(i)}\right\}$) are given:

3 $$ J\left(\theta \right)=\sum \limits_i\log \left({p}_{main}\left({y}^{(i)}|{x}^{(i)},\theta \right)\right)+\sum \limits_i\log \left({p}_{aux}\left({y}_{aux}^{(i)}|{x}^{(i)},\theta \right)\right) $$

### Post-processing

After the disease mentions are recognized with MCNN, two simple post-processing steps are carried out to boost the final performance. First, when the tagged label sequence is ill-legal (which is defined in Table 1), the corresponding labels will be set to “O”. Second, if the full name of an abbreviation is recognized as a disease mention, all the abbreviation will be also labeled as a disease. The full names and the corresponding abbreviations were extracted from the test set using an in-house tool. Since both post-processing strategies are not task-specific, they can be applied to other NER problems easily.

**Table 1:** The legal and ill-legal sequences

Sequence	Legal	Ill-legal
B, O, …	*	
B, B, O, …	*	
B, I, O, …	*	
B, I, B, I, O,…	*	
O, I, O, …		*
O, I, B, O, …		*

### Experimental results and discussion

#### Experimental datasets and settings

MCNN model was implemented using Keras (https://keras.io/) that is a minimalist, highly modular neural networks library written in Python. We trained and tested our model on a GPU of Nvidia Tesla k20. Using the settings discussed in this section, the model training cost about 1.5 h for the NCBI corpus and 2 h for the CDR corpus.

We validated the effectiveness of MCNN by applying it to two corpora containing both mention-level and concept-level annotations: the NCBI Disease corpus [4] and the BioCreative V Chemical Disease Relation task (CDR) corpus [18]. Overall statistics for each dataset are provided in Table 2. The NCBI Disease corpus consists of 793 Medline abstracts separated into training (593), development (100) and test (100) subsets. The NCBI Disease corpus is annotated with disease mentions, using concept identifiers from either MeSH or OMIM.

**Table 2:** The statistics of CDR and NCBI corpora

Corpus		Training	Development	Test
CDR	Abstract	500	500	500
	Mention	4182	4244	4424
NCBI	Abstract	593	100	100
	Mention	5145	787	960

The BioCreative V Chemical Disease Relation (CDR) corpus consists of 1500 Medline abstracts, separated into training (500), development (500) and test (500) sets. We reconstructed a development set by separating 100 abstracts from the original development set and put the rest 400 abstracts into the training set. The CDR corpus is annotated with concept identifiers from MeSH.

In addition, several hyper-parameters need to be determined in MCNN. The hyper-parameters and their values used in our experiments are shown in Table 3. Due to time constrains it is infeasible to do a grid-search across the full hyper-parameter space. Therefore, the hyper-parameters were tuned on the development sets by random search. In addition, the learning rates were determined using the default value of the SGD strategy provided by Keras (https://keras.io/optimizers), as it can achieve pretty excellent performance. For the NER tasks on NCBI corpus and CDR corpus, the hyper-parameter values are almost the same, except the number of stacked convolutional layers.

**Table 3:** The hyper-parameters and corresponding values

Hyper-parameter	Value
Input context window size	13 (NCBI = CDR)
Word-level embedding dimension	200 (NCBI = CDR)
Character-level embedding dimension	20 (NCBI = CDR)
Lexicon feature embedding dimension	5 (NCBI = CDR)
Character-level CNN’s window size	3 (NCBI = CDR)
Character-level CNN’s filters number	20 (NCBI = CDR)
Word-level CNN’s window size	3 (NCBI = CDR)
Word-level CNN’s filters number	100 (NCBI = CDR)
Word-level Convolutional layers size	3 (NCBI); 4 (CDR)

“BIO” tagging scheme was adopted in our experiments, where “B” stands for beginning, “I” for intermediate and “O” for outsider or other. Similar to other systems, MCNN was evaluated using the balanced F-score = (2PR)/(P + R), where P denotes the precision and R denotes the recall. The NER measure is mention level which requires the predicted span to exactly match the annotated span.

### Performance comparison with other methods

The performance comparison between our MCNN method and other state-of-the-art methods is shown in Table 4. As can be seen from Table 4, MCNN achieves the state-of-the-art performance on both NCBI and CDR corpora.

**Table 4:** Performance comparisons on NCBI and CDR corpora

Corpus	Method	P	R	F
NCBI	BANNER [4]	83.80	80.00	81.80
	Bi-LSTM + WE [14]	84.87	74.11	79.13
	**MCNN**	**85.08**	**85.26**	**85.17**
	MCNN*	83.74	83.03	83.39
CDR	HITSZ_CDR [5]	88.68	85.23	86.93
	Lee et al.’s [6]	87.34	83.75	85.51
	CRD-DNER [31]	79.49	73.58	76.42
	**MCNN**	**88.20**	**87.46**	**87.83**

Note. MCNN* is the version of removing the lexicon feature embedding and the post-processing step

On the NCBI corpus, we compared the performance of MCNN with that of BANNER [4] and Bi-LSTM + WE [14]. BANNER is a CRF based bio-entity recognition model, which utilizes the general linguistic, orthographic, syntactic dependency features, etc. It achieves the state-of-the-art result on NCBI corpus. Compared with BANNER, our method obtains higher F-score (85.17 vs. 81.80) with much less feature engineering. Besides the word-level and the character-level embeddings, in our method, only the lexical feature embedding is extracted using MEDIC. It indicates that the MCNN can learn useful features automatically while the conventional CRF based methods rely heavily on task-specific feature engineering. Similar to our method, Bi-LSTM + WE, which was reported in ACL 2016, also utilizes a deep learning method to solve the disease NER problem. Bi-directional LSTM (BLSTM) is employed in Bi-LSTM + WE while our method employs the CNNs and a CRF layer is stacked on top of the BLSTM in Bi-LSTM + WE to decode labels of a sentence jointly while our method utilizes MLS to capture the correlation information. Besides the current word, using MLS, the previous and the next words’ labels are predicted simultaneously. In addition, MCNN utilizes lexicon feature embedding and post-processing strategy to improve the final performance. To make it more comparable, the lexicon feature embedding and the post-processing step are removed from our method (the method is called MCNN*) before comparing with Bi-LSTM + WE. Then it is found that MCNN*, the removed version, still performs better than Bi-LSTM + WE (F-scores of 83.39 vs. 79.13). The reason may be that Bi-LSTM + WE treats NER as a sentence level sequence tagging problem while NER is not a complex sentence level problem but a simple word level classification problem. As known to all, using a complicated model to learn an easy problem will over-fit easily. Thus, MCNN may handle the NCBI disease NER problem better than Bi-LSTM + WE as it treats NER as a simple word level problem which can avoid the over-fitting problem.

On the CDR corpus, the performance of HITSZ_CDR [5], Lee et al.’s [6] and CRD-DNER [31] were compared with that of MCNN. CDR corpus [18] was created for automatic chemical disease relation (CDR) extraction challenge which includes two subtasks: disease named entity recognition (DNER) and chemical-induced disease (CID) relation extraction. We compared our method with HITSZ_CDR and Lee et al.’s, as their results rank first and second, respectively, in the DNER subtask. HITSZ_CDR [5] employs two sequence labeling methods (CRF and structure support vector machine) to tag an input sequence separately. Then, a linear SVM, as the meta-classifier, is used to check whether a mention recognized by any one of the two previous methods is correct or not. HITSZ_CDR extracts rich hand-crafted features: n-gram words, n-gram POSs, sentence length, words’ affixes, words’ shapes, words’ orthographical information, dictionary feature, word embedding feature, etc. Lee et al. also proposed a CRF based method that needs multiple well-designed features. Besides the normal features like linguistic, orthographic, etc., terminology and vowel features were extracted. As can be seen, mass of feature engineering is employed in both HITSZ_CDR and Lee et al.’s approach to achieve the higher performance. On the contrast, the MCNN method needs little feature engineering where all the three embeddings are tuned automatically during the training process. Finally, the MCNN approach outperforms HITSZ_CDR and Lee et al.’s (F-scores of 87.83 vs. 86.93 and 85.51), which proves again that MCNN can learn useful features automatically.

We also compared the MCNN with CRD-DNER [31] as it employs a deep learning method (RNN) as well. As shown in Table 4, CRD-DNER performs poor compared with MCNN. The reason may be that it only employs the simple RNN but not the LSTM which has been proved to be more effective than simple RNN [14]. And it does not take into consideration the correlations between neighboring labels. What’s more, it doesn’t integrate character-level representation that is widely used to model a word’s morphological information.

### The effect analysis of each feature/strategy

Besides the fundamental word embedding based CNN model, following four features/strategies are adopted in our method to boost our final performance. 1) Character-level: character-level representation is employed to extract morphological information (like the prefix or suffix information) of a word. 2) Lexicon: MEDIC resource is utilized to extract lexicon feature embedding. 3) MLS: besides the target label, the previous and the next labels are predicted in auxiliary through the training process to capture the correlation information between neighboring labels. 4) Post-processing: the illegal label sequences (defined in Table 1) and the missed disease mentions (represented as abbreviations) are regulated in the post-processing step. To evaluate the effectiveness of these features/strategies, the corresponding experiments were conducted with MCNN: we remove a feature or a strategy each time and then calculate the F-score and the corresponding decrease compared with the one before it is removed.

As can be seen from Table 5, the lexicon feature embedding contributes most to our method on both NCBI and CDR corpora, as removing the lexicon feature embedding decreases the F-scores on two corpora by 1.73 and 2.64, respectively. Since MEDIC is a complete disease vocabulary, it may help to recall many disease mentions that are not covered by the training set. In addition, the MLS strategy also plays a key role in our method (decreasing the F-scores by 0.98 and 1.16 on NCBI and CDR corpora, respectively). MLS strategy must has avoided generating many ill-legal label sequences. However, some ill-legal label sequences are still remained, and they will be removed in the post-processing step. In addition, the missed disease mentions’ abbreviations will be retrieved in post-processing step as well. Finally, removing the whole post-processing step causes the decrease of F-scores by 0.33 and 1.15 on NCBI and CDR corpora, respectively. Compared with the features/strategies above, char-level representation contributes least to MCNN, as the F-scores are decreased by 0.71 and 0.53 on NCBI and CDR corpora, respectively, after removing it. The disease mentions’ simple word-formation may restrict the character-level representation’s ability which is created to extract word’s morphological information. Intuitively, the character-level representation will be brought into full-play in chemical or protein NER problem whose word-formation is much complex. For example, the chemicals are often represented as the forms like N-[4-(5-nitro-2-furyl)-2-thiazolyl]-formamide, alpha,beta-methylene adenosine-5′-triphosphate, pralidoxime-2-chloride, etc. and the proteins like IFN-alpha, senescence-associated beta-galactosidase, p53, ET-3, etc. As can be seen, chemical and protein mentions often show a complex structure by mixing of letters, digits and symbols while it happens rarely for disease mentions.

**Table 5:** The effect analysis of each feature/strategy

Corpus	Feature/strategy	P	R	F-score	Δ
NCBI	None	85.08	85.26	85.17	–
	Character-level	84.50	84.41	84.46	0.71
	Lexicon	83.53	83.35	83.44	1.73
	MLS	83.97	84.41	84.19	0.98
	Post-processing	84.84	84.84	84.84	0.33
CDR	None	88.20	87.46	87.83	–
	Character-level	87.25	87.35	87.30	0.53
	Lexicon	85.48	84.90	85.19	2.64
	MLS	87.18	86.17	86.67	1.16
	Post-processing	86.67	86.69	86.68	1.15

Notes. Δdenotes the corresponding F-score decrease when a strategy or a feature is removed

## Conclusions

In this paper, we present a novel convolutional neural network based disease NER architecture (MCNN). The concatenation of the word-level, the character-level and the lexicon feature embeddings is fed to the CNN model. Then a CNN-based classifier is learned to recognize the disease mentions in the texts. Finally, MCNN achieves the state-of-the-art performance on both NCBI and CDR corpora.

The main contributions of our work can be summarized as follows: 1. Little feature engineering is needed in MCNN as the word-level embedding, the character-level embedding and the lexicon feature embedding can be tuned automatically during the training process. 2. Multiple label strategy is introduced to capture the correlation information between labels in neighborhoods and it has been proved to be effective and efficient.

MCNN exhibits promising results for disease NER in the biomedical texts. Nevertheless, the performance still has much room for improvement. In the future work, we will further improve the MCNN model to achieve better performance.
